# Correlation between p-STAT3 overexpression and prognosis in lung cancer: A systematic review and meta-analysis

**DOI:** 10.1371/journal.pone.0182282

**Published:** 2017-08-10

**Authors:** Mengting Tong, Jing Wang, Nanyu Jiang, Hongming Pan, Da Li

**Affiliations:** Department of Medical Oncology, Sir Run Run Shaw Hospital, Zhejiang University School of Medicine, Hangzhou, Zhejiang, China; National Cancer Center, JAPAN

## Abstract

**Objective:**

Previous studies have shown the correlation between p-STAT3 overexpression and prognosis in a variety of human tumors. However, their correlation in lung cancer remains controversial. We performed a systematic review and meta-analysis to explore the correlation between p-STAT3 overexpression and prognosis in lung cancer patients.

**Methods:**

We searched PubMed, Embase, Web of Science, CNKI, VIP, and WanFang Data to identify relevant studies. Two reviewers independently screened the literature search results, extracted data, and assessed the methodological quality of the included studies. Then, meta-analysis was performed by using Review Manager 5.3 and STATA 14 software. A random-effect model was employed to evaluate all related pooled results. Statistical heterogeneity of each study was assessed by I^2^. Publication bias was determined by funnel plot and the Begg’s or Egger’s tests.

**Results:**

Eventually, 13 studies were included in present meta-analysis. Among these 13 studies, 8 studies were associated with the overall survival of lung cancer and 10 studies with other clinicopathological characteristics. The results of this meta-analysis suggested that p-STAT3 overexpression may be a poor prognosis biomarker in lung cancer (HR: 1.23; 95% CI: 1.04–1.46; P = 0.02). In terms of other clinicopathological characteristics, p-STAT3 overexpression was more frequent to advanced TNM stages ranging from III to IV (OR: 1.92; 95% CI: 1.13–3.27; P = 0.02) and lymphatic node metastasis (OR: 1.81; 95% CI: 1.20–2.72; P = 0.004). But, it was not associated with tumor differentiation (OR: 0.82; 95% CI: 0.44–1.53; P = 0.54).

**Conclusion:**

p-STAT3 overexpression has significant correlation with poorer overall survival of lung cancer patients, as well as with more advanced TNM stages and lymph node metastasis. Thus, it may serve a biomarker for poor prognosis in lung cancer. Nevertheless, our findings should be confirmed by large prospective studies.

## Introduction

Cancer is considered a major public health problem worldwide, with an estimated 1,688,780 new cases and 600,920 deaths in 2017 according to the latest global statistics [1]. Lung cancer is one of the most common malignant tumors with the second highest incidence rate among all tumors. It is also the first leading cause of cancer-related deaths in both sexes. The 5-year survival of localized lung cancer patients can reach 55.2%. However, more than 50% of the lung cancer patients are diagnosed in advanced stages with a 5-year survival of less than 20%, despite the rapid development of diagnosis and treatment [2–3]. Due to the specific living environment and habits in China [4–6], the incidence and mortality rate of lung cancer are higher than the global average rates [7]. In recent years, increasingly more research has been devoted to investigation of on the use of molecular predictors for prognosis in cancer patients. Furthermore, such predictors could be utilized for the selection of potential therapeutic targets. At present, large-scale randomized controlled trials (RCTs) elucidating the mechanism of gene panel and cell cycle progression of effective molecular predictors are underway [8]. Now, an urgent need exists to identify critical molecular predictors of lung cancer progression because of its high morbidity and mortality.

The family of signal transducer and activator of transcription (STAT) proteins in the cytoplasm include seven transcription factors: STAT1, STAT2, STAT3, STAT4, STAT5a, STAT5b, and STAT6 [9]. Among them, STAT3 has been recognized as one of the key factors for tumors formation [10–11]. It is a known fact that there are two active forms of STAT3: one of them is a type of dimer (SH2), which is stable and difficult to degrade [12], and the second one is phospho-STAT3 (p-STAT). In the cytoplasm, STAT3 is phosphorylated to p-STAT3 by Janus kinases (*JAKs*). Before the phosphorylation of STAT3, *JAK* is activated by anaplastic lymphoma kinase (*ALK*) and growth factor receptors, including the epidermal growth factor receptor (*EGFR*), platelet-derived growth factor (*PDGF*), and macrophage colony-stimulating factor (*CSF1*). Then, p-STAT3 enters into the nucleus and promotes tumor cell proliferation, drug resistance, or suppresses tumor cell apoptosis [13–17].

Recent studies have demonstrated that p-STAT3 overexpression is associated with poorer prognosis in patients with gastric cancer [18], colorectal carcinoma [19], pancreatic cancer [20], and lung cancer [21]. However, no association between p-STAT3 overexpression and lung cancer development has been found in some recent studies [22–24]. Therefore, we conducted this updated meta-analysis to explore the correlation between p-STAT3 overexpression and the overall survival of lung cancer patients, as well as other clinicopathological characteristics.

## Materials and methods

This meta-analysis was performed in accordance with the Preferred Reporting Items for Systematic Reviews and Meta-Analyses (PRISMA) guideline (S1 File).

### Search strategy

We searched PubMed, Embase, Web of Science, CNKI, VIP, and WanFang Data to identify relevant studies (the ending date of our search was November 1, 2016). The following words (in Chinese) were used for retrieval of relevant studies: lung cancer, pSTAT3, phospho-STAT3, *etc*. In addition, the following retrieval combinations (in English) were utilized: (lung neoplasm OR small cell lung carcinoma OR non-small cell lung carcinoma OR NSCLC OR SCLC OR lung cancer) AND (STAT3 OR STAT3 transcription factor OR signal transducer and activator of transcription 3 OR STAT3 protein OR pSTAT3 OR phospho-STAT3 OR phosphorylated signal transducer and activator of transcription 3 OR phosphorylated STAT3 transcription factor)

### Inclusion criteria

The following inclusion criteria were employed: (1) the study sample was from clinically diagnosed lung cancer patients; (2) an immunohistochemical (IHC) method was used to detect p-STAT3 expression; (3) hazard ratios (HRs) with a 95% confidence interval (CI) were used to evaluate the correlation between p-STAT3 overexpression and the overall survival of lung cancer patients, or the Kaplan-Meier survival curves were used for the assessment; (4) the study provided sufficient data to calculate the odds ratios (ORs), which were utilized to evaluate the correlation between p-STAT3 overexpression and the clinicopathological characteristics in lung cancer patients; (5) if similar results were reported in more than one journal, we accepted only those from the most recent or the most complete study; (6) if the results reported in the identified publications were published in different languages, we accepted only one of them; (7) all investigations included were in Chinese or English, and all articles in Chinese were published in core journals in China.

### Data extraction

All titles and abstracts identified in the initial search were independently screened by two researchers (MTT and JW), and studies that did not satisfy the inclusion criteria were excluded. Subsequently, the full-text articles were reviewed, and all available data were extracted. The extracted information included: (1) the title of the paper, the name of first author, publication year, and the number of samples; (2) patient age, gender, follow-up time, the location of p-STAT3 expression, and the cut-off value of p-STAT3; (3) tumor information: TNM stage, lymph node metastasis, differentiation, *etc*. All data were cross-checked by two researchers. In cases of disagreement, consensus was achieved through evaluation by a third reviewer (NYJ). If the study information was incomplete or unclear, we contacted the author to collect as accurate information as possible.

### Quality assessment

Two authors (MTT and JW) independently assessed the quality of the included studies using the Newcastle-Ottawa Scale (NOS score) [25]. A score ≥ 6 was consider to indicate high-quality articles.

### Statistical analysis

HRs with 95% CI were used to evaluate the correlation of p-STAT3 overexpression with the overall survival of lung cancer patients. If HRs with 95% CI were not available in the original article but with Kaplan-Meier survival curves, all results were calculated, and Kaplan-Meier survival curves were read by Engauge Digitizer version 4.1 software (http://sourceforge.net/projects/digitizer) and Jayne F Tierney table (http://www.biomedcentral.com/content/supplementary/1745-6215-8-16-S1.xls).) ORs with 95%CI were used to evaluate the correlation of p-STAT3 overexpression with clinicopathological characteristics in lung cancer patients. In this meta-analysis, all results including HRs and ORs were pooled by the random-effects model. I^2^ was used to assess statistical heterogeneity. If I^2^>50%, heterogeneity was considered to exist among all included studies, and we conducted a subgroup analysis to investigate its possible source. If I^2^< 50%, heterogeneity among all included studies was regarded as insignificant, and data were directly pooled. To access the stability of our meta-analysis results, we conducted a sensitivity analysis by omitting individual studies in turn and transforming the random effect model into the fixed-effects model. Visual inspection of funnel plots for overall survival was conducted, and the Begg’s or Egger’s tests were used to determine the potential publication bias. Further, a meta-analysis was conducted by using Review Manager (version 5.3, Cochrane Collaboration, Copenhagen, Denmark) and STATA software (version 14 StataCorp, Texas, USA). The P-values were two-sided and values < 0.05 were considered statistically significant.

## Results

### Study selection

**Fig 1:**
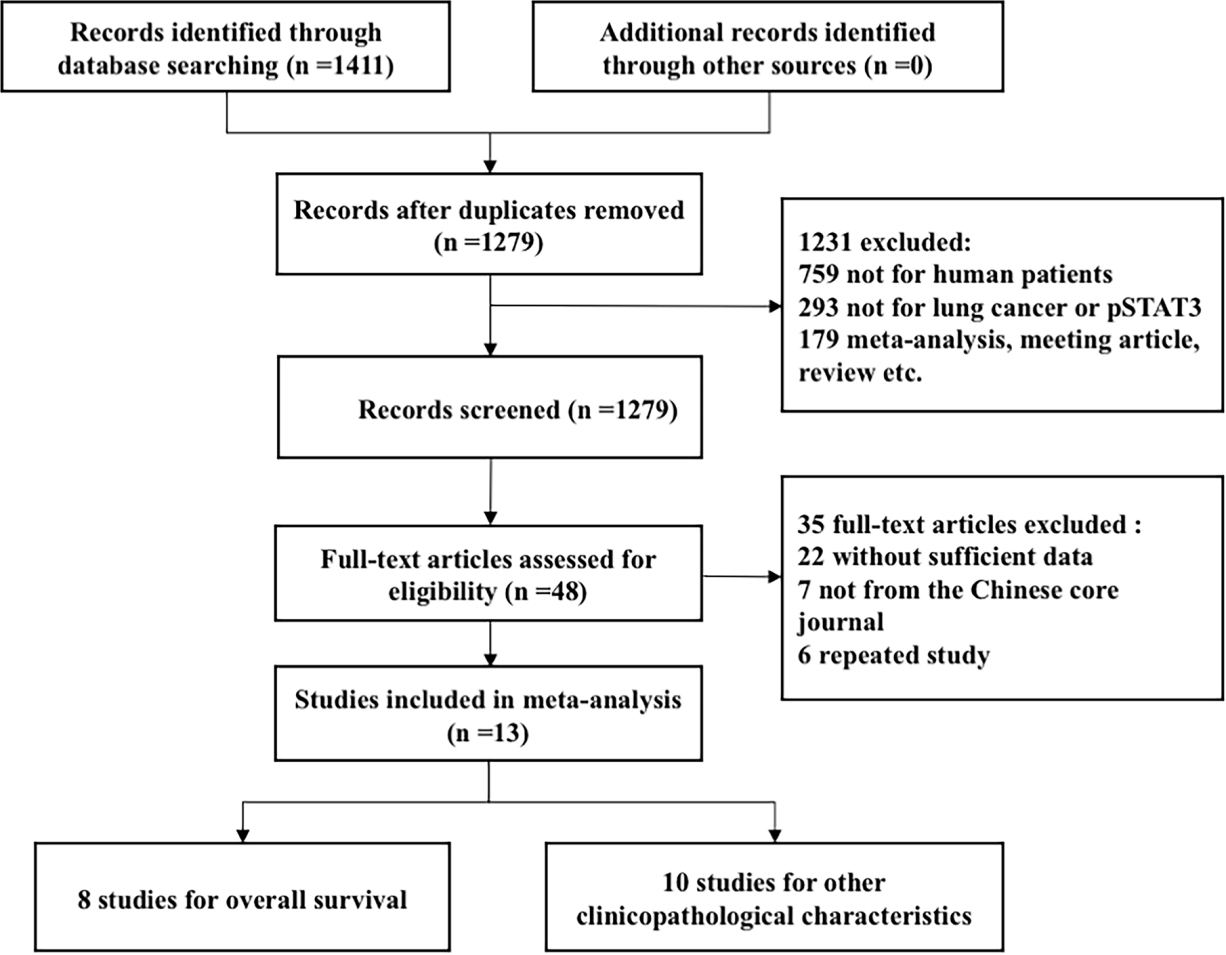
Flowchart of the literature search.

A total of 1,411 relevant studies were identified by using the search strategy described earlier, of which 1,279 studies were included by reviewing their titles and abstracts. Of them, we selected 48 studies that were eligible for a full-text review. Finally, a total of 13 eligible studies were included in this meta-analysis (flowchart in Fig 1, Fig 1 in the S1 File). Eight of these studies were analyzed to determine the correlation of p-STAT3 overexpression with the overall survival of lung cancer patients [15,22,24,26–30]. And ten studies were subjected to the meta-analysis of clinicopathological characteristics [31–35].

### Study characteristics

The basic characteristics of the 1,848 individuals included in the 13 eligible studies are summarized in Table 1. The number of patients included in each examination ranged from 60 to 303, and the follow-up period spanned from 0 to 146 months. Only one of these 13 studies involved small lung cancer cases [22], and another one enrolled only patients with lung adenocarcinoma [31].

**Table 1:** Characteristics and results for 13 included studies.

Author	Publication year	Country	No.	Gender (M/F)	Media age(Y)	Media Follow-up	N/C	Positive (%)	Cut-off value	Stage I-II/III-IV	Grade I-II/III	LN (+)	OS	HR estimate	NOS Score
Zhao[22]	2012	China	128	66/62	—	15.4m	N	48.43	—	77/51	44/15	69	OS	HR	8
Yu[26]	2015	China	82	48/34	59	36.3m	N	59.76	—	38/44	—	61	OS	HR	8
Wang[27]	2011	China	208	128/80	59.8	67m	N	46.15	25%	158/50	132/76	142	OS	HR	8
Wang[33]	2012	China	59	32/27	57.6	—	N	52.54	10%	46/13	44/15	28	—	—	—
Yang[35]	2012	China	87	58/29	58	—	N	59.34	10%	—	59/28	44	—	—	—
Cortas[29]	2007	USA	145	61/84	70	—	N	37.31	>5%	123/22	—	58	OS	HR	7
Achcar[32]	2007	USA	303	181/122	—	>5y	N	60.53	33%	244/59	—	—	—	_	—
Zhao[30]	2011	China	68	38/30	59.4	—	—	68.96	50%	27/41	29/39	41	OS	HR	8
Haura[15]	2005	USA	176	97/79	69	37m	—	—	—	—	—	_	OS	K-M	7
Jiang[24]	2016	China	194	122/72	59	41m	N	46.20	—	127/67	130/64	87	OS	HR	7
Mukohara[34]	2003	Japan	60	45/15	67.5	8.4y	—	58.33	>5%	40/20	35/25	—	—	—	_
Kim[31]	2010	Korea	162	93/69	62	67m	—	68.96	25%	126/36	103/43	50	OS	—	—
van Cruijsen[28]	2009	USA	176	127/49	64.5	—	N	70.70	50%	141/35	—	—	OS	K-M	7

M, male; F, female; N, nucleus; C, cytoplasm; No., patients number; LN, Lymph node metastasis; Positive, percentage of pSTAT3 positive cells; K-M, Kaplan-Meier survival carves; OS, Overall survival; "—", not mentioned.

### Quality assessment

NOS score was used to determine the quality of the included studies. Since several studies concerning other clinicopathological characteristics supplied only partially useful information, we assessed the NOS score only of the studies investigating the overall survival of lung cancer patients. Thus, among the 8 studies assessed, 4 studies had a NOS score of 8, and 4 studies scored 7 (Table 2).

**Table 2:** Quality assessment of each included study according to the Newcastle-Ottawa Scale.

study	Publication year	Selection	Comparability	Outcome	NOS score
Haura[15]	2005	☆☆	☆☆	☆☆☆	7
Zhao[22]	2012	☆☆☆	☆☆	☆☆☆	8
Jiang[24]	2016	☆☆	☆☆	☆☆☆	7
Yu[26]	2015	☆☆☆	☆☆	☆☆☆	8
Wang[27]	2011	☆☆☆	☆☆	☆☆☆	8
van Cruijsen[28]	2009	☆☆	☆☆	☆☆☆	7
Cortas[29]	2007	☆☆	☆☆	☆☆☆	7
Zhao[30]	2011	☆☆☆	☆☆	☆☆☆	8

### Meta-analysis results

#### p-STAT3 overexpression and overall survival

**Fig 2:**
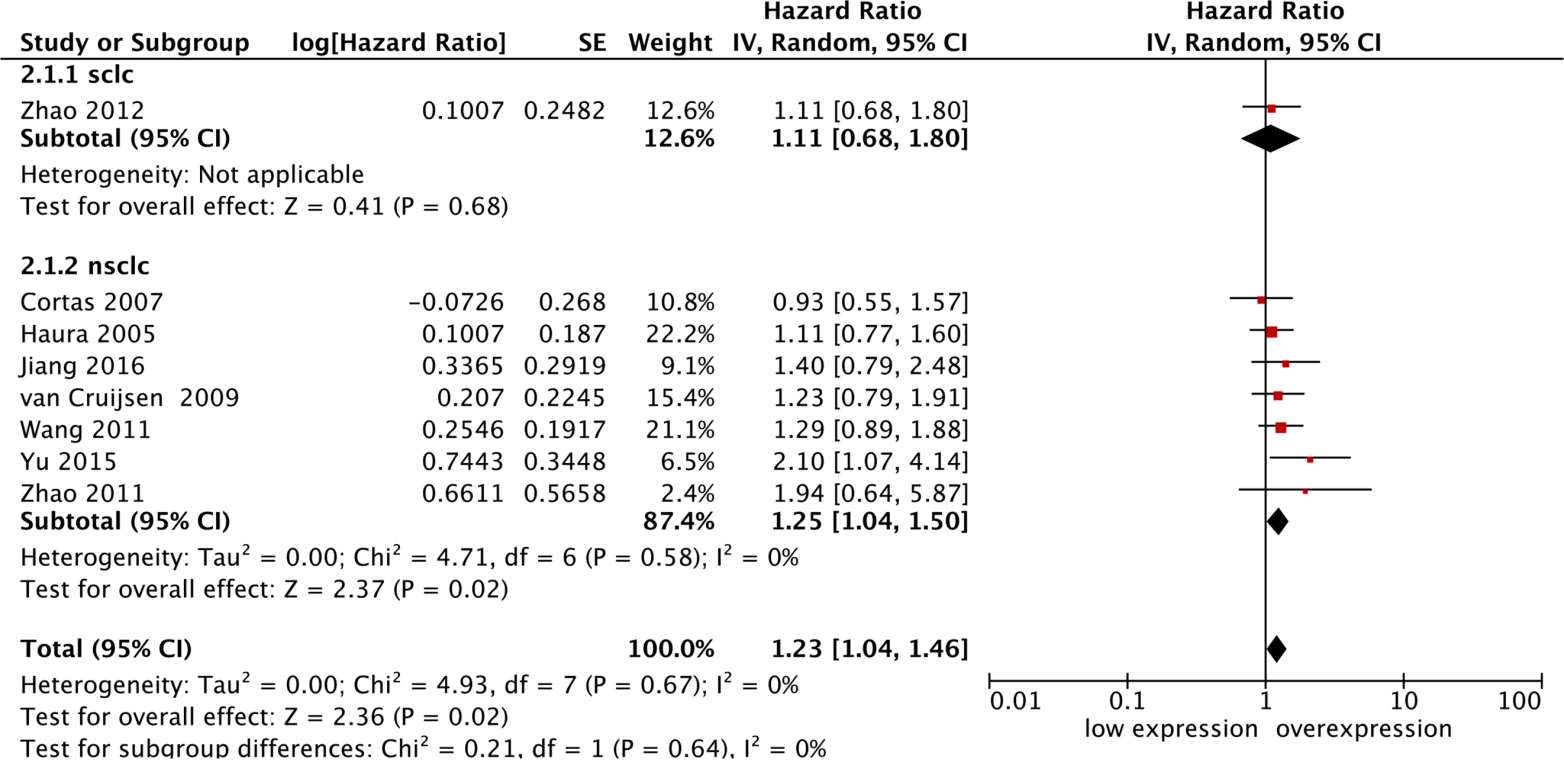
Forest plot for the association of p-STAT3 overexpression with overall survival in lung cancer patients.

In this meta-analysis of eight studies, we found that p-STAT3 was overexpressed in lung cancer patients with poorer overall survival. The pooled HR was 1.23 (95% CI: 1.04–1.46; I^2^ = 0%), and the difference was statistically significant (P = 0.02) (Fig 2).

#### p-STAT3 overexpression and TNM stage

**Fig 3:**
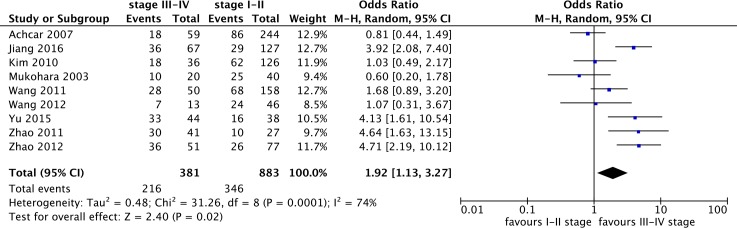
Forest plot for the association of p-STAT3 overexpression with TNM stage in lung cancer patients.

Nine studies with a total of 1,624 enrolled individuals were included in the meta-analysis of p-STAT3 overexpression and TNM stage. The results indicated that p-STAT3 overexpression was more frequent for clinical TNM stages ranging from III to IV, and the pooled OR was 1.92 (95% CI: 1.13–3.27) with statistically significant difference (Fig 3). Heterogeneity existed among all included studies (I^2^ = 74%), thus, a subgroup analysis based on ethnicity was conducted. The pooled OR in China was 3.04 (95% CI: 1.93–4.79; P<0.00001; I^2^ = 44%), and heterogeneity decreased considerably from 74% to 44%. Therefore, we can conclude that ethnicity is a significant source of heterogeneity in this meta-analysis.

#### p-STAT3 overexpression and lymph node metastasis

**Fig 4:**
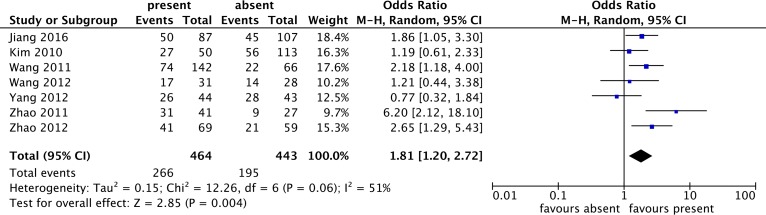
Forest plot for the association of p-STAT3 overexpression with lymph node metastasis in lung cancer patients.

Seven studies, enrolling 464 patients with lymphatic node metastasis and 443 patients without lymphatic node metastasis, were included in the present meta-analysis of the association between p-STAT3 overexpression and lymph node metastasis. Our findings revealed that p-STAT3 overexpression was more frequent for the lymphatic node metastasis group, whose pooled OR was 1.81(95%CI: 1.20–2.72) as determined by the random-effects model with statistically significant differences (Fig 4). Heterogeneity was present among all included studies (I^2^ > 50%), and a subgroup analysis based on the number of sample was conducted. The pooled OR in the large sample size (n≥100) was 1.88 (95% CI: 1.37–2.59; P < 0.0001; I^2^ = 0%), and heterogeneity was reduced substantially from 51% to 0%.

#### p-STAT3 overexpression and tumor differentiation

**Fig 5:**
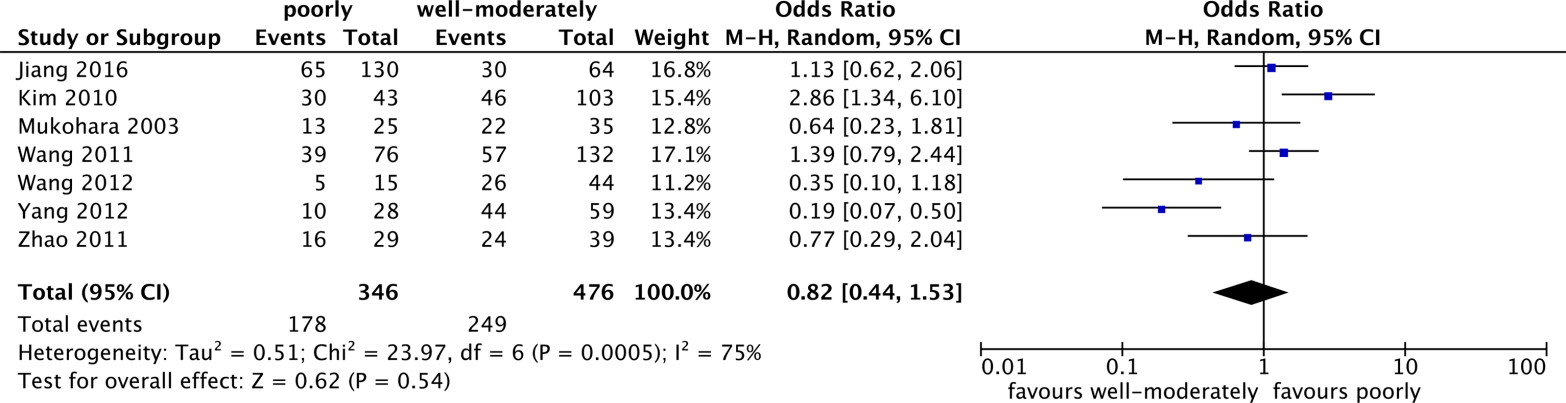
Forest plot for the association of p-STAT3 overexpression with tumor differentiation in lung cancer patients.

Seven studies with 822 patients were included in the meta-analysis of the relation between p-STAT3 overexpression and differentiation in lung cancer patients. However, there was no statistically significant difference in the well- moderately and poorly differentiation (OR = 0.54; 95% CI: 0.55–4.43) (Fig 5).

In addition, we also pooled ORs between p-STAT3 overexpression and pathological types (adenocarcinoma and squamous carcinoma), smoking history, and patient ages, but, there was no significant association (data not shown).

### Sensitivity analyses

Identical results were obtained in the cases when the fixed effects model was used, including those of the pooled HRs and ORs. However, omitting individual studies in turn contributed to achieving a significant influence on the combined HRs (Table 3), but no influence on the pooled ORs (data not shown).

**Table 3:** Sensitivity analysis for p-STAT3 overexpression with overall survival.

Study omitted	Publication year	HR	95%CI	p-value	*I*^2^
Haura[15]	2005	1.27	1.04–1.54	0.02	0%
Zhao[22]	2012	1.25	1.04–1.50	0.02	0%
Jiang[24]	2016	1.22	1.01–1.46	0.03	0%
Yu[26]	2015	1.19	0.99–1.42	0.06	0%
Wang[27]	2011	1.22	1.00–1.48	0.05	0%
van Cruijsen[28]	2009	1.23	1.02–1.48	0.03	0%
Cortas[29]	2007	1.27	1.06–1.53	0.01	0%
Zhao[30]	2011	1.22	1.02–1.45	0.03	0%

### Publication bias

**Fig 6:**
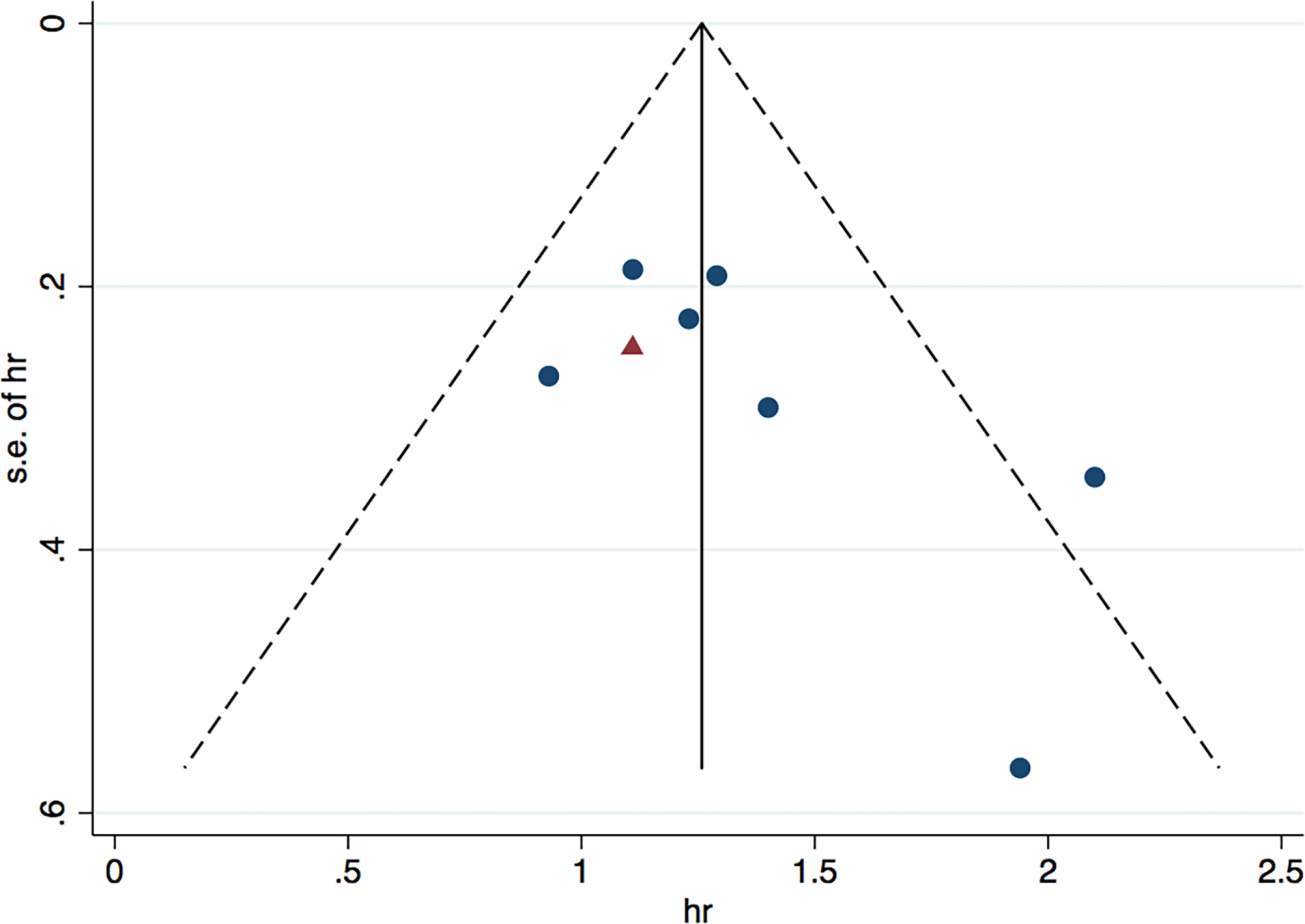
Funnel plot for p-STAT3 overexpression with overall survival in lung cancer patients.

Funnel plot was used to estimate the potential publication bias for the association between p-STAT3 expression and the overall survival (Fig 6). The shape of the funnel plot was asymmetrical. Because the number of the included studies was small, we conducted only the Egger’s test, which revealed no evidence of publication bias (P = 0.097). Although the Egger’s test suggested that there might have been no potential publication bias, we comprehensively analyzed the information and speculated that the small sample size and the exclusion of some unpublished studies might have affected the results.

## Discussion

In 1986, *Mountain* firstly described the TNM stage [36]. Later, after many revisions, the concept was adopted by the American Joint Committee on Cancer (AJCC) and the Union International Cancer Center (UICC). From then onwards, the TNM stage has been continually used in clinical theory and practice. However, we found that the prognosis of lung cancer patients might be different in despite of the presence of identical TNM stages. In recent years, an association between some molecular markers and the prognosis of lung cancer has been detected in a large number of investigations. For example, *Koh*, *et al*. found that PD-1 overexpression in lung cancer patients led to poorer overall survival and progression-free survival [37]. In addition, *Wang et al*. evidenced that the decreased expression of miR-133a in lung cancer patients was related to a worse prognosis, including a more advanced TNM stage and increased lymph node metastasis [38]. Furthermore, the research of *Takamizawa*, *et al*. discovered that let-7 microRNA overexpression in lung cancer postoperative patients was associated with a considerably shorter survival time [39]. Moreover, different associations between p-STAT3 overexpression and tumors have been found by many scientists. For example, a considerable number of meta-analyses has shown the presence of a certain association between p-STAT3 overexpression and the poorer prognosis of patients with solid tumors [40], digestive tract tumors [41], gastric cancer [42], colorectal cancer [43], and lung cancer [21]. However, opposite results were obtained in the meta-analysis conducted by *Kong et al*. [44]. Therefore, no unified conclusion has been reached on the association between p-STAT3 overexpression and tumor prognosis.

As early as 1999, *Golob et al*. found that p38 mitogen-activated protein (*MAP*) kinase completely inhibited p-STAT3 expression [45]. In addition, other studies have also shown that it inhibits tumor cell growth and distant metastasis, but promotes tumor cell apoptosis by inhibiting STAT3 phosphorylation, the transcription and replication of DNA mediated by p-STAT3 [46–47]. At present, a number of phase I clinical trials on p-STAT3 inhibitors (https://clinicaltrials.gov/) are underway. It is noteworthy that *Shou et al*. also found that progression-free survival of the group of lung cancer patients with p-STAT3 overexpression was significantly shorter (9 months vs 26 months, P < 0.05) after the application of *EGFR-TKI* treatment [48]. Previous studies have suggested that p-STAT3 might be a potential prognostic marker or therapeutic target. Therefore, we conducted this meta-analysis to elucidate the association between p-STAT3 overexpression and the overall survival of lung cancer patients, as well as between p-STAT3 overexpression and other clinicopathological characteristics.

Our meta-analysis, including three new negative results, was conducted based on the study of *Xu et al*. *published* in 2014 [21]. In spite of the identical result obtained, we considered that our study was not an updated meta-analysis but a revised version. Furthermore, we drew a primary conclusion that p-STAT3 overexpression was associated with poorer overall survival of lung cancer patients (HR = 1.23; 95% CI: 1.04–1.46; P = 0.02). In terms of clinicopathological characteristics, p-STAT3 overexpression was more frequent to the group of patients with TNM stages ranging from III to IV, and patients with lymphatic node metastasis. Although, the results of the meta-analysis of concerning overall survival are not stable, p-STAT3 can still be regarded as a biomarker indicator for poor prognosis in lung cancer patients.

Of course, our meta-analysis has certain limitations: (1) the cut-off value of p-STAT3 was different in each of the studies; (2) a single method for detection of p-STAT3 expression was used. The reliability and stability of the results is related to the researchers involved and the levels of the respective research centers; (3) the location of p-STAT3 expression was unclear; although most studies specified that p-STAT3 was located in the nucleus, its location was not described clearly in some of them. As is well known, only a small amount of the cytoplasmic expression of p-STAT3 might interfere with results; (4) since no specific data were provided in some studies, especially concerning HR with 95% CI, we extracted this information from the Kaplan-Meier survival curves, which might have caused some discrepancy with the real data; (5) direct exclusion of the specific data in some studies might have affected the final results; (6) the sample size of some studies was small, and even the sources of patients, patients age, gender, follow-up time, *etc*., may be factors that might have influenced the pooled results.

In conclusion, p-STAT3 overexpression is associated with poorer overall survival of lung cancer patients, as well as with a more advanced TNM grade and lymph node metastasis. Thus, it may serve as a biomarker indicator for poor prognosis in lung cancer patients. Nevertheless, this conclusion should be confirmed by large prospective studies with long-term follow-up.

## Supporting information

S1 File Flowchart of the literature search. (DOC)

S2 File Completed 2009 PRISMA checklist. (DOC)

S3 File Search strategy. (DOC)

## References

[1] Siegel RL, Miller KD, Jemal A. Cancer Statistics, 2017. CA Cancer J Clin. 2017;67(1):7–30. doi: 10.3322/caac.21387 . 28055103 10.3322/caac.21387

[2] Hirsch FR, Scagliotti GV, Mulshine JL, Kwon R, Curran WJ Jr., Wu YL, et al Lung cancer: current therapies and new targeted treatments. Lancet. 2017;389(10066):299–311. doi: 10.1016/S0140-6736(16)30958-8 . 27574741 10.1016/S0140-6736(16)30958-8

[3] Howlader N, Noone AM, Krapcho M, Miller D, Bishop K, Altekruse SF, et al. SEER Cancer Statistics Review, 1975–2013, National Cancer Institute. http://seer.cancer.gov/csr/1975_2013/, based on November 2015 SEER data submission, posted to the SEER web site, April 2016.

[4] Thun MJ, Hannan LM, Adams-Campbell LL, Boffetta P, Buring JE, Feskanich D, et al Lung cancer occurrence in never-smokers: an analysis of 13 cohorts and 22 cancer registry studies. PLoS Med 2008;5(9):e185 doi: 10.1371/journal.pmed.0050185 18788891 10.1371/journal.pmed.0050185 PMC2531137

[5] Lam WK, White NW, Chan-Yeung MM. Lung cancer epidemiology and risk factors in Asia and Africa. Int J Tuberc Lung Dis 2004;8(9):1045–57. 15455588

[6] Gao YT, Blot WJ, Zheng W, Ershow AG, Hsu CW, Levin LI, et al Lung cancer among Chinese women. Int J Cancer 1987;40(5):604–9. 2824385 10.1002/ijc.2910400505

[7] Chen WPMD, Zheng RMPH, Baade PD, M. P. H., Zhang S, B. MedSc, et al Cancer statistics in china, 2015. Ca: A Cancer Journal for Clinicians, 66(2), 115 Retrieved from http://search.proquest.com/docview/1790494671?accountid=15198;2(66):115. 10.3322/caac.21338 26808342

[8] Zheng Y, Bueno R. Commercially available prognostic molecular models in early stage lung cancer a review of the Pervenio Lung RS and Myriad myPlan Lung Cancer tests. 2016. 10.1586/14737159.2015.1028371 25896578

[9] Darnell JJ. STATs and gene regulation. Science 1997;277(5332):1630–5. 9287210 10.1126/science.277.5332.1630

[10] Bowman T, Garcia R, Turkson J, Jove R. STATs in oncogenesis. Oncogene 2000;19(21):2474–88. doi: 10.1038/sj.onc.1203527 10851046 10.1038/sj.onc.1203527

[11] Bromberg J, Darnell JJ. The role of STATs in transcriptional control and their impact on cellular function. Oncogene 2000;19(21):2468–73. doi: 10.1038/sj.onc.1203476 10851045 10.1038/sj.onc.1203476

[12] Novak U, Ji H, Kanagasundaram V, Simpson R, Paradiso L. STAT3 forms stable homodimers in the presence of divalent cations prior to activation. Biochem Biophys Res Commun 1998;247(3):558–63. doi: 10.1006/bbrc.1998.8829 9647732 10.1006/bbrc.1998.8829

[13] Vogelstein B, Kinzler KW. Cancer genes and the pathways they control. Nat Med 2004;10(8):789–99. doi: 10.1038/nm1087 15286780 10.1038/nm1087

[14] Kanda N, Seno H, Konda Y, Marusawa H, Kanai M, Nakajima T, et al STAT3 is constitutively activated and supports cell survival in association with survivin expression in gastric cancer cells. Oncogene 2004;23(28):4921–9. doi: 10.1038/sj.onc.1207606 15077160 10.1038/sj.onc.1207606

[15] Haura EB, Zheng Z, Song L, Cantor A, Bepler G. Activated epidermal growth factor receptor-Stat-3 signaling promotes tumor survival in vivo in non-small cell lung cancer. Clin Cancer Res 2005;11(23):8288–94. doi: 10.1158/1078-0432.CCR-05-0827 16322287 10.1158/1078-0432.CCR-05-0827

[16] Król M, Pawłowski KM, Dolka I, Musielak O, Majchrzak K, Mucha J, et al Density of Gr1-positive myeloid precursor cells, p-STAT3 expression and gene expression pattern in canine mammary cancer metastasis. Veterinary Research Communications 2011;35(7):409–423. doi: 10.1007/s11259-011-9489-3 21713436 10.1007/s11259-011-9489-3 PMC3165193

[17] Duan ZF, Foster R, Bell DA, Mahoney J, Wolak K, Vaidya A, et al Signal transducers and activators of transcription 3 pathway activation in drug-resistant ovarian cancer. CLINICAL CANCER RESEARCH 2006;12(17):5055–5063. doi: 10.1158/1078-0432.CCR-06-0861 16951221 10.1158/1078-0432.CCR-06-0861

[18] Yakata Y, Nakayama T, Yoshizaki A, Kusaba T, Inoue K, Sekine I. Expression of p-STAT3 in human gastric carcinoma: significant correlation in tumour invasion and prognosis. Int J Oncol 2007;30(2):437–42. 17203226

[19] Kusaba T, Nakayama T, Yamazumi K, Yakata Y, Yoshizaki A, Inoue K, et al Activation of STAT3 is a marker of poor prognosis in human colorectal cancer. Oncol Rep 2006;15(6):1445–51. 16685378

[20] Wormann SM, Song L, Ai J, Diakopoulos KN, Kurkowski MU, Gorgulu K, et al Loss of P53 Function Activates JAK2-STAT3 Signaling to Promote Pancreatic Tumor Growth, Stroma Modification, and Gemcitabine Resistance in Mice and Is Associated With Patient Survival. Gastroenterology 2016;151(1):180–193.e12. doi: 10.1053/j.gastro.2016.03.010 27003603 10.1053/j.gastro.2016.03.010

[21] Xu YH, Lu S. A meta-analysis of STAT3 and phospho-STAT3 expression and survival of patients with non-small-cell lung cancer. Eur J Surg Oncol 2014;40(3):311–7. doi: 10.1016/j.ejso.2013.11.012 24332948 10.1016/j.ejso.2013.11.012

[22] Zhao X, Sun X, Li XL. Expression and clinical significance of STAT3, P-STAT3, and VEGF-C in small cell lung cancer. Asian Pac J Cancer Prev 2012;13(6):2873–7. 22938476 10.7314/apjcp.2012.13.6.2873

[23] Zhang W, Pal SK, Liu X, Yang C, Allahabadi S, Bhanji S, et al Myeloid clusters are associated with a pro-metastatic environment and poor prognosis in smoking-related early stage non-small cell lung cancer. PLoS One 2013;8(5):e65121 doi: 10.1371/journal.pone.0065121 23717691 10.1371/journal.pone.0065121 PMC3663795

[24] Jiang R, Wang X, Jin Z, Li K. Association of Nuclear PIM1 Expression with Lymph Node Metastasis and Poor Prognosis in Patients with Lung Adenocarcinoma and Squamous Cell Carcinoma. J Cancer 2016;7(3):324–34. doi: 10.7150/jca.13422 26918046 10.7150/jca.13422 PMC4747887

[25] Stang A. Critical evaluation of the Newcastle-Ottawa scale for the assessment of the quality of nonrandomized studies in meta-analyses. Eur J Epidemiol 2010;25(9):603–5. doi: 10.1007/s10654-010-9491-z 20652370 10.1007/s10654-010-9491-z

[26] Yu Y, Zhao Q, Wang Z, Liu XY. Activated STAT3 correlates with prognosis of non-small cell lung cancer and indicates new anticancer strategies. Cancer Chemother Pharmacol 2015;75(5):917–22. doi: 10.1007/s00280-015-2710-2 25735252 10.1007/s00280-015-2710-2

[27] Wang M, Chen GY, Song HT, Hong X, Yang ZY, Sui GJ. Significance of CXCR4, phosphorylated STAT3 and VEGF-A expression in resected non-small cell lung cancer. Exp Ther Med 2011;2(3):517–522. doi: 10.3892/etm.2011.235 22977534 10.3892/etm.2011.235 PMC3440730

[28] van Cruijsen H, Ruiz MG, van der Valk P, de Gruijl TD, Giaccone G. Tissue micro array analysis of ganglioside N-glycolyl GM3 expression and signal transducer and activator of transcription (STAT)-3 activation in relation to dendritic cell infiltration and microvessel density in non-small cell lung cancer. BMC Cancer 2009;9:180 doi: 10.1186/1471-2407-9-180 19519895 10.1186/1471-2407-9-180 PMC2705377

[29] Cortas T, Eisenberg R, Fu P, Kern J, Patrick L, Dowlati A. Activation state EGFR and STAT-3 as prognostic markers in resected non-small cell lung cancer. Lung Cancer 2007;55(3):349–55. doi: 10.1016/j.lungcan.2006.11.003 17161498 10.1016/j.lungcan.2006.11.003

[30] Zhao M, Gao FH, Wang JY, Liu F, Yuan HH, Zhang WY, et al JAK2/STAT3 signaling pathway activation mediates tumor angiogenesis by upregulation of VEGF and bFGF in non-small-cell lung cancer. Lung Cancer 2011;73(3):366–74. doi: 10.1016/j.lungcan.2011.01.002 21333372 10.1016/j.lungcan.2011.01.002

[31] Kim HS, Park YH, Lee J, Ahn JS, Kim J, Shim YM, et al Clinical impact of phosphorylated signal transducer and activator of transcription 3, epidermal growth factor receptor, p53, and vascular endothelial growth factor receptor 1 expression in resected adenocarcinoma of lung by using tissue microarray. Cancer 2010;116(3):676–85. doi: 10.1002/cncr.24748 20052735 10.1002/cncr.24748

[32] Achcar RO, Cagle PT, Jagirdar J. Expression of activated and latent signal transducer and activator of transcription 3 in 303 non-small cell lung carcinomas and 44 malignant mesotheliomas: possible role for chemotherapeutic intervention. Arch Pathol Lab Med 2007;131(9):1350–60. doi: 10.1043/1543-2165(2007)131[1350:EOAALS]2.0.CO;2 17824789 10.5858/2007-131-1350-EOAALS

[33] Wang RJ, Zhang JZ, Wang P. [Expression of pSTAT3 in non-small cell lung cancer and its clinical significance]. Xi Bao Yu Fen Zi Mian Yi Xue Za Zhi 2012;28(3):288–90. 22394639

[34] Mukohara T, Kudoh S, Yamauchi S, Kimura T, Yoshimura N, Kanazawa H, et al Expression of epidermal growth factor receptor (EGFR) and downstream-activated peptides in surgically excised non-small-cell lung cancer (NSCLC). Lung Cancer 2003;41(2):123–30. 12871775 10.1016/s0169-5002(03)00225-3

[35] Yang Q, Shen SS, Zhou S, Ni J, Chen D, Wang G, et al STAT3 activation and aberrant ligand-dependent sonic hedgehog signaling in human pulmonary adenocarcinoma. Exp Mol Pathol 2012;93(2):227–36. doi: 10.1016/j.yexmp.2012.04.009 22554932 10.1016/j.yexmp.2012.04.009

[36] Mountain CF. A new international staging system for lung cancer. Chest 1986;89(4 Suppl):225S–233S. 3514171 10.1378/chest.89.4_supplement.225s

[37] Koh J, Jang JY, Keam B, Kim S, Kim MY, Go H, et al EML4-ALK enhances programmed cell death-ligand 1 expression in pulmonary adenocarcinoma via hypoxia-inducible factor (HIF)-1alpha and STAT3. Oncoimmunology 2016;5(3):e1108514 doi: 10.1080/2162402X.2015.1108514 27141364 10.1080/2162402X.2015.1108514 PMC4839370

[38] Wang Y, Li J, Chen H, Mo Y, Ye H, Luo Y, et al Down-regulation of miR-133a as a poor prognosticator in non-small cell lung cancer. Gene 2016;591(2):333–7. doi: 10.1016/j.gene.2016.06.001 27282282 10.1016/j.gene.2016.06.001

[39] Takamizawa J, Konishi H, Yanagisawa K, Tomida S, Osada H, Endoh H, et al Reduced expression of the let-7 microRNAs in human lung cancers in association with shortened postoperative survival. CANCER RESEARCH 2004;64(11):3753–3756. doi: 10.1158/0008-5472.CAN-04-0637 15172979 10.1158/0008-5472.CAN-04-0637

[40] Wu P, Wu D, Zhao L, Huang L, Shen G, Huang J, et al Prognostic role of STAT3 in solid tumors: a systematic review and meta-analysis. Oncotarget 2016;7(15):19863–83. doi: 10.18632/oncotarget.7887 26959884 10.18632/oncotarget.7887 PMC4991424

[41] Li MX, Bi XY, Huang Z, Zhao JJ, Han Y, Li ZY, et al Prognostic Role of Phospho-STAT3 in Patients with Cancers of the Digestive System: A Systematic Review and Meta-Analysis. PLoS One 2015;10(5):e0127356 doi: 10.1371/journal.pone.0127356 26024373 10.1371/journal.pone.0127356 PMC4449159

[42] He S, Liao G, Liu Y, Huang L, Kang M, Chen L. Overexpression of STAT3/pSTAT3 was associated with poor prognosis in gastric cancer: a meta-analysis. Int J Clin Exp Med 2015;8(11):20014–23. 26884913 PMC4723758

[43] Ji K, Zhang M, Chu Q, Gan Y, Ren H, Zhang L, et al The Role of p-STAT3 as a Prognostic and Clinicopathological Marker in Colorectal Cancer: A Systematic Review and Meta-Analysis. PLoS One 2016;11(8):e0160125 doi: 10.1371/journal.pone.0160125 27504822 10.1371/journal.pone.0160125 PMC4978497

[44] Kong H, Zhang Q, Zeng Y, Wang H, Wu M, Zheng T, et al Prognostic significance of STAT3/phosphorylated-STAT3 in tumor: a meta-analysis of literatures. Int J Clin Exp Med 2015;8(6):8525–39. 26309504 PMC4537978

[45] Gollob JA, Schnipper CP, Murphy EA, Ritz J, Frank DA. The functional synergy between IL-12 and IL-2 involves p38 mitogen-activated protein kinase and is associated with the augmentation of STAT serine phosphorylation. J Immunol 1999;162(8):4472–81. 10201984

[46] Yue P, Turkson J. Targeting STAT3 in cancer: how successful are we? Expert Opin Investig Drugs 2009; 18(1):45–56. doi: 10.1517/13543780802565791 19053881 10.1517/13543780802565791 PMC2610472

[47] Huang W, Dong Z, Chen Y, Wang F, Wang CJ, Peng H, et al Small-molecule inhibitors targeting the DNA-binding domain of STAT3 suppress tumor growth, metastasis and STAT3 target gene expression in vivo. Oncogene 2016;35(6):783–92. doi: 10.1038/onc.2015.215 26073084 10.1038/onc.2015.215

[48] Shou J, You L, Yao J, Xie J, Jing J, Jing Z, et al Cyclosporine A sensitizes human non-small cell lung cancer cells to gefitinib through inhibition of STAT3. Cancer Lett 2016; 379(1):124–33. doi: 10.1016/j.canlet.2016.06.002 27264264 10.1016/j.canlet.2016.06.002

