# Supplementary material for: Correlation between p-STAT3 overexpression and prognosis in lung cancer: A systematic review and meta-analysis
Source: PLoS One. 2017 Aug 10;12(8):e0182282. doi: 10.1371/journal.pone.0182282 (PMC5552221; doi:10.1371/journal.pone.0182282)
Supplement: S1 File — (DOC) [file pone.0182282.s001.doc]

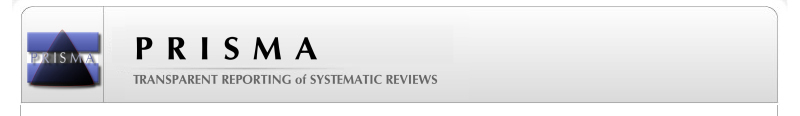
**PRISMA 2009 Flow Diagram**

**Screening**

**Included**

**Eligibility**

**Identification**

Records identified through database searching
(n =1411)

Additional records identified through other sources
(n =0)

Records after duplicates removed
(n =1279)

Records screened
(n =1279)

1231 excluded:
759 not for human patients

293 not for lung cancer or pSTAT3

179 meta-analysis, meeting article, review etc

Full-text articles assessed for eligibility
(n =48)

35 full-text articles excluded:

22 without sufficient data

7 not from the Chinese core journal

6 repeated study

6

Studies included in meta-analysis
(n =13)

8 studies for overall survival

10 studies for other clinicopathological characteristics
