# Supplementary material for: Correlation between p-STAT3 overexpression and prognosis in lung cancer: A systematic review and meta-analysis
Source: PLoS One. 2017 Aug 10;12(8):e0182282. doi: 10.1371/journal.pone.0182282 (PMC5552221; doi:10.1371/journal.pone.0182282)
Supplement: S3 File — (DOC) [file pone.0182282.s003.doc]

**Pubmed:**

#1 Search ((((((((STAT3) OR STAT3 transcription factor) OR (signal transducer and activator of transcription 3)) OR STAT3 protein) OR pSTAT3) OR phospho-STAT3) OR (phosphoralated signal transducer and activator of transcription 3)) OR phosphoralated STAT3 transcription factor) OR protein stat3 (n=16587)

#2 Search (((((((((lung cancer) OR pulmonary cancer) OR lung tumor) OR lung neoplasm) OR lung tumour) OR superior sulcus tumor) OR small cell lung cancer) OR bronchial small cell cancer) OR bronchial non small cell cancer) OR non small cell lung cancer) OR SCLC) OR NSCLC (n=310046)

#3 #1 AND #2 AND (humans [Mesh]) (n=589)

**Web of Science:**

#1 (n=19660)

TOPIC: (STAT3) OR TOPIC: (STAT3 transcription factor) OR TOPIC: (signal transducer and activator of transcription 3) OR TOPIC: (STAT3 protein) OR TOPIC: (pSTAT3) OR TOPIC: (phospho-STAT3) OR TOPIC: (phosphoralated signal transducer and activator of transcription 3) OR TOPIC: (phosphoralated STAT3 transcription factor)

Indexes=SCI-EXPANDED, SSCI, A&HCI, CPCI-S, ESCI, CCR-EXPANDED, IC Timespan=All years

#2 (n=279742)

TOPIC: (lung cancer) OR TOPIC: (pulmonary cancer) OR TOPIC: (lung tumor) OR TOPIC: (lung neoplasm) OR TOPIC: (lung tumour) OR TOPIC: (superior sulcus tumor) TOPIC: (small cell lung cancer) TOPIC: (bronchial small cell cancer) TOPIC: (bronchial non small cell cancer) TOPIC: (non small cell lung cancer) TOPIC: (SCLC) TOPIC: (NSCLC)

Indexes=SCI-EXPANDED, SSCI, A&HCI, CPCI-S, ESCI, CCR-EXPANDED, IC Timespan=All years

#3 (n=553)

#1 AND #2

Indexes=SCI-EXPANDED, SSCI, A&HCI, CPCI-S, ESCI, CCR-EXPANDED, IC Timespan=All years

**Embase:**

#1 'human'/exp OR 'human' OR 'lung cancer' OR 'lung carcinogenesis'/exp OR 'lung carcinogenesis' OR 'lung carcinoma'/exp OR 'lung carcinoma' OR nsclc OR sclc OR 'non-small cell lung cancer'/exp OR 'small cell lung cancer'/exp AND (stat3 OR pstat3 OR'stat3 protein'/exp) AND ('progression free survival'/exp OR 'odds ratio'/exp OR 'hazard ratio'/exp)

#2 'human'/exp OR ('diseases'/exp OR 'disease' OR 'diseases' OR 'disorder' OR 'illness' OR 'sickness') AND (stat3 OR pstat3) AND (mpfs OR os OR 95% ci)

#3 #1 AND #2 (n=229)

**Full list of the Chinese search strategy**

Chinese retrieval words: lung cancer, pSTAT3 and phospho-STAT3

Timespan=All years

Source type= core journals

CNKI (n=8)

VIP （n=12）

WanFang Data (n=20)

**中文检索策略**

检索关键词：肺癌 pSTST3 磷酸化STAT3

发表年限：全部年份

来源类别：核心期刊

中国知网 （n=8）

维普数据库 （n=12）

万方数据库 （n=20）
